# PR Interval as a Novel Therapeutic Target of Ivabradine Therapy—Prognostic Impact of Ivabradine-Induced PR Prolongation in Heart Failure Patients

**DOI:** 10.3390/jcm13020510

**Published:** 2024-01-16

**Authors:** Riona Yamamoto, Naoya Kataoka, Teruhiko Imamura, Toshihide Izumida, Koichiro Kinugawa

**Affiliations:** Second Department of Internal Medicine, University of Toyama, 2630 Sugitani, Toyama 930-0194, Japanteimamu@med.u-toyama.ac.jp (T.I.); kinugawa@med.u-toyama.ac.jp (K.K.)

**Keywords:** heart rate, ivabradine, PR interval, trans-mitral flow pattern

## Abstract

Background: Ivabradine reduces heart rate by inhibiting the “funny current” expressed on the sinoatrial node and improves mortality and morbidity in patients with systolic heart failure and sinus tachycardia. The funny current is known to be expressed also on the atrioventricular node according to experimental studies. However, the impact of ivabradine on PR interval remained unknown. Methods: Patients with a left ventricular ejection fraction of less than 50% who received 1 month of ivabradine were screened. Electrocardiographic and echocardiographic data, particularly concerning heart rate, the PR interval, and trans-mitral flow pattern, were collected at baseline and 1-month follow-up. The primary endpoint was defined as the composite of cardiovascular death and hospital readmission for worsening heart failure following ivabradine administration. Results: In the cohort of 29 enrolled patients (median age: 66 years, 62% male), the median baseline heart rate was 86 beats per minute and the median PR interval was 168 milliseconds. Following ivabradine administration, a significant decrease of 20 beats per minute in the heart rate and a significant increase of 24 milliseconds in the PR interval were observed. The truncated interval of the A-wave, detected in the trans-mitral flow, consistently demonstrated a negative correlation with the PR interval both before and after the administration of ivabradine. During a median of 1.8 years of follow-up, six patients reached the primary endpoint. A combination of heart rate reduction and PR prolongation following ivabradine administration, both of which were independent factors associated with the primary endpoint (*p* < 0.05 for both), was associated with greater freedom from the primary endpoint compared with either/neither of them (*p* = 0.002). Conclusions: Ivabradine seems to prolong PR interval, which is a novel surrogate marker of favorable clinical outcomes in patients with systolic heart failure. This effect may be associated with the dynamics of the trans-mitral flow pattern, in conjunction with heart rate and the PR interval. Clinical implications of PR interval-guided ivabradine therapy remains the future concern.

## 1. Introduction

Ivabradine is a crucial medical therapy for heart failure with systolic dysfunction when patients exhibit a high heart rate [1]. It inhibits the ‘funny current (I_f_)’, which includes mixed sodium and potassium inward current permeability, primarily expressed in the sinoatrial node [2]. This channel is activated during phase 4 of the action potential, leading to an acceleration of diastolic depolarization [2]. Thus, ivabradine can reduce heart rate through mechanisms distinct from other negative chronotropic agents, such as beta-blockers, calcium channel blockers, or sodium channel blockers, all of which have negative inotropic effects. By reducing heart rate, ivabradine therapy increases cardiac output, facilitates reverse remodeling, and improves mortality and morbidity in patients with systolic heart failure.

While extensive research has been conducted on heart rate and the prognosis of patients with systolic heart failure, there has not been as much detailed research on electrocardiographic changes by ivabradine as one might expect. Recent experimental literature demonstrated that I_f_ is expressed also on the atrioventricular node [3]. Previous clinical trials have demonstrated the utility of ivabradine in reducing heart rate during atrial fibrillation, instead of sinus tachycardia [4,5]. In experimental models, ivabradine reduced atrioventricular conduction in a rate-dependent manner [6]. While the PR interval gradually extends concomitantly with a decrease in heart rate, ivabradine’s impact on atrioventricular conduction beyond the physiological response to a reduced heart rate has remained insufficiently elucidated in a clinical context [7].

**Figure 1:**
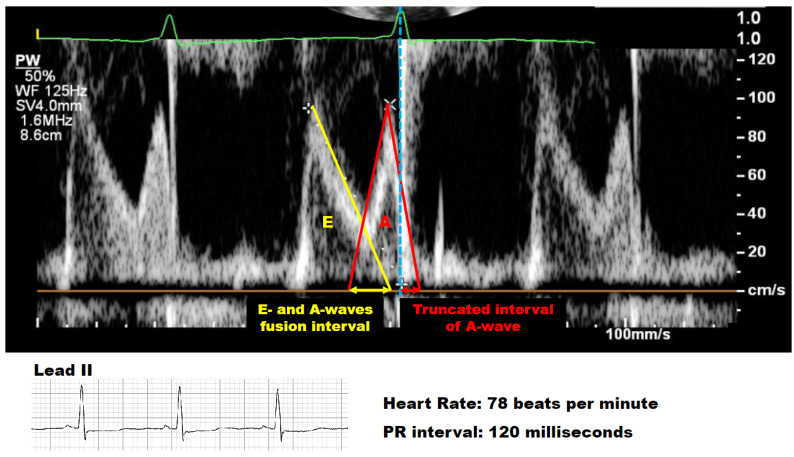
How to assess the fusion of E- and A-waves and the truncated A-wave intervals. In a representative trans-mitral flow pattern with a heart rate of 78 beats per minute and a PR interval of 120 milliseconds in lead II, the terminal E-wave and the initial A-wave exhibited fusion, while the terminal A-wave was truncated as a result of mitral valve closure. The yellow arrow designates the interval of fusion between the E- and A-waves; the red arrow indicates the truncated interval of the A-wave. The blue dotted line corresponds to the timing of the R-wave on the electrocardiogram (green line), suggesting the closure of the mitral valve.

Our group previously reported that favorable outcomes become evident with the optimization of the echocardiographic trans-mitral E-wave and A-wave overlap following a reduction in heart rate by ivabradine therapy [8]. Interestingly, the PR interval has a crucial association with trans-mitral filling velocities [9,10]. A short PR interval demonstrates truncation of the A-wave by the early closure of the mitral valve (Figure 1) [10]. Therefore, a hypothesis had arisen that not only the decrease in heart rate but also the prolongation of the PR interval by ivabradine may be a key to successful ivabradine therapy by increasing cardia output and facilitating reverse remodeling.

Hence, the present study was conducted to address the following two clinical questions: (1) Does ivabradine affect the PR interval on surface electrocardiograms? (2) Does PR prolongation have a favorable prognostic impact in patients with systolic heart failure?

## 2. Methods

### 2.1. Study Population

Patients with systolic heart failure who received ivabradine at least for 1 month between December 2019 and June 2023 were retrospectively screened. The following criteria were applied for subject inclusion: (1) age of 20 years or older, (2) a diagnosis of symptomatic heart failure (New York Heart Association class II, III, or IV) with a left ventricular ejection fraction of less than 50%, and (3) availability of electrocardiograms recorded both before and at 1 month after ivabradine administration, indicating sinus rhythm with a resting heart rate of 75 bpm or higher as measured by surface electrocardiogram. The exclusion criteria were as follows: (1) patients with persistent atrial fibrillation, (2) patients who had undergone cardiac intervention within the past three months, (3) patients who were lost to follow-up within a month following ivabradine administration, (4) patients who had their beta-blocker dose adjusted during follow-up, which may influence electrocardiographic parameters, and (5) patients who had received right ventricular pacing through cardiac implantable electronic devices, due to the difficulty of assessing the PR interval. The current study was carried out in accordance with the Declaration of Helsinki and received approval from the Institutional Review Board at the University of Toyama.

### 2.2. Patient Characteristics

The clinical characteristics, encompassing demographic information and laboratory data, were retrieved from the electronic medical records before ivabradine administration. The electrocardiographic parameters measured in lead II and echocardiographic parameters related to Doppler trans-mitral flow velocity were evaluated within one week before the administration of ivabradine and one month after administration by two independent investigators, Y.R. and N.K. The fusion of E- and A-waves was assessed as a hemodynamic impact factor. Furthermore, the interval from the R-wave onset in the surface electrocardiogram to the termination of the A-wave, indicating a truncated atrial kick, was also assessed (Figure 1) [11]. To assess the pure atrioventricular nodal effects of ivabradine, the interval of (PR interval–P-wave duration) was also examined, in addition to the assessment of P-wave duration.

### 2.3. Clinical Outcomes

Heart rate reduction and PR interval prolongation resulting from ivabradine were assessed both before and one month after administration. The primary endpoint was defined as the composite of cardiovascular death and hospital readmission for worsening heart failure. The impact of electrocardiographic changes, particularly PR prolongation, on the primary endpoint, was investigated.

### 2.4. Statistical Analysis

The data were presented as medians with interquartile ranges. Categorical data were expressed as numbers and percentages. Bivariate correlation analysis was performed utilizing Pearson’s correlation coefficient. Cox proportional hazard model analyses were performed to assess whether clinical characteristics were associated with the primary endpoints after administration. Subsequently, multivariable analyses were conducted, adjusting for clinical variables with *p*-values less than 0.05 in the univariable analyses, to investigate independently predictive factors. Kaplan–Meier survival curves were generated, and the log-rank test was employed to assess group differences. Data analysis was carried out using JMP version 14 (SAS, Cary, NC, USA).

## 3. Results

### 3.1. Clinical Characteristics

A total of 29 patients (median age: 66 years, 62% male) were enrolled in the study (Table 1). Approximately half of the enrolled subjects exhibited concurrent hypertension and dyslipidemia, with three patients undergoing hemodialysis. About 40% of the subjects manifested ischemic heart disease, and the median left ventricular ejection fraction was 38%. Due to constraints in the registration period, the administration rate of sodium-glucose transport protein 2 inhibitors was only 34%, and guideline-directed medical therapy, comprising the four anti-heart failure medications, was attained in 28% of the enrolled subjects. The median trans-mitral flow pattern demonstrated an impaired relaxation pattern, characterized by an E/A ratio of 0.9. Additionally, the median fusion of E- and A-waves was 68 milliseconds (msec). 

### 3.2. Trajectory of Electrocardiographic Parameters

The baseline heart rate exhibited a median (interquartile range) of 86 (79–101) bpm; PR interval of 168 (140–182) msec; P-wave duration of 104 (91–116) msec; interval of (PR–P-wave) of 64 (38–85) msec; QRS width of 105 (86–125) msec; and corrected QT interval by Bazzet’s formula of 469 (443–513) msec.

**Figure 2:**
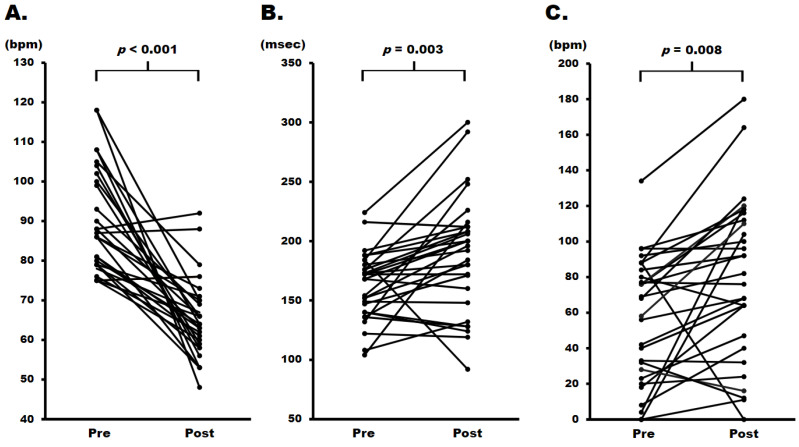

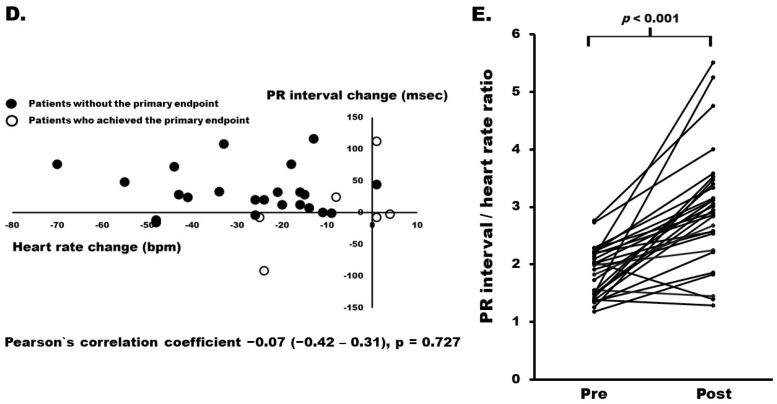
Changes of electrocardiographic parameters following ivabradine administration. (**A**) Heart rate; (**B**) PR interval; (**C**) Interval of (PR interval–P-wave duration); (**D**) correlation between the alterations in heart rate and PR interval following ivabradine administration. The whiteout tags indicate patients who achieved the primary endpoint during the follow-up period. (**E**) Changes of heart rate-adjusted PR interval before and after ivabradine administration; bpm; beats per minute. msec; milliseconds.

Changes in these parameters following one month of ivabradine administration at a median dose of 5 mg twice daily are illustrated in Figure 2. After administration, a significant reduction in heart rate (decrease of 20 [10–38] bpm, *p* < 0.001; Figure 2A) and an increase in the PR interval (prolongation of 24 [−2–46] msec, *p* = 0.003; Figure 2B) were observed. Additionally, the interval of (PR–P-wave) significantly prolonged by 20 [−4–46] msec (*p* = 0.008, Figure 2C). On the contrary, there were no significant changes in P-wave duration (decrease of 4 [10–−2] msec, *p* = 0.163), QRS width (increase of 4 [−10–12] msec, *p* = 0.794), or corrected QT interval (increase of 9 [−25–28] msec, *p* = 0.791). While the majority exhibited PR prolongation concurrent with a decrease in heart rate, the changes in heart rate and PR interval did not demonstrate a statistically significant correlation (Pearson’s correlation coefficient: −0.07, *p* = 0.727, Figure 2D). To mitigate the impact of heart rate reduction on PR interval prolongation, the PR interval adjusted by heart rate was also evaluated. The PR interval to heart rate ratio significantly increased one month after ivabradine administration compared to that before (*p* < 0.001, Figure 2E).

### 3.3. Association between Electrocardiographic and Echocardiographic Data

**Figure 3:**
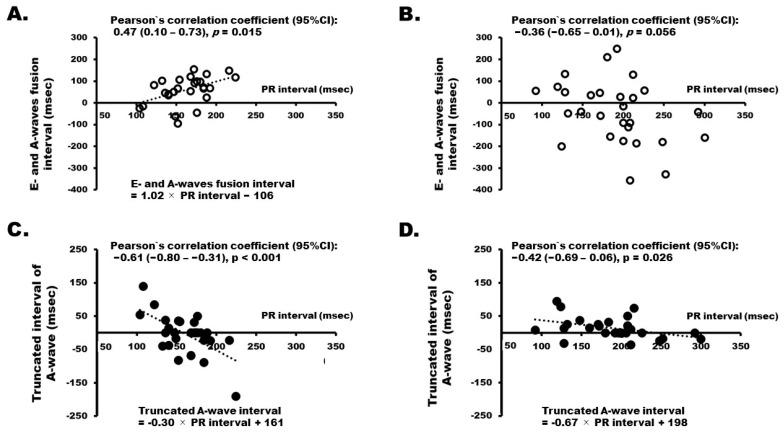
Correlation between electrocardiographic parameters and fusion or truncated intervals of trans-mitral flow. (**A**) The correlation between E- and A-waves fusion interval and PR interval before ivabradine administration; (**B**) The correlation between E- and A-waves fusion interval and PR interval after ivabradine administration; (**C**) The correlation between truncated interval of A-wave and PR interval before ivabradine administration; (**D**) The correlation between truncated interval of A-wave and PR interval after ivabradine administration; CI indicates confidence interval. Other abbreviations are the same as Figure 2.

In the context of trans-mitral flow, the heart rate did not consistently exhibit a statistically significant correlation with the fusion interval between E- and A-waves, both before and after ivabradine administration (Pearson’s correlation coefficient (95% confidence interval) 0.05 [−0.33–0.43], *p* = 0.789 and 0.23 [−0.16–0.56], *p* = 0.237, respectively). A positive correlation was observed in the PR interval before administration (*p* = 0.015, Figure 3A); however, this significance diminished after administration (*p* = 0.056, Figure 3B). Notably, the truncated interval of the A-wave showed a consistently significant negative correlation with the PR interval both before and after ivabradine administration (*p* < 0.001 and *p* = 0.026, respectively; Figure 3C,D); however, the heart rate did not exhibit such a correlation (Pearson’s correlation coefficient 0.03 [−0.35–0.40], *p* = 0.887 and 0.33 [−0.15–0.62], *p* = 0.088, respectively).

### 3.4. Predictors of the Primary Endpoints

Over a median follow-up period of 1.8 years, four patients experienced cardiovascular death. A total of four patients faced worsening heart failure necessitating hospitalization. Notably, two of these patients were readmitted exclusively.

**Figure 4:**
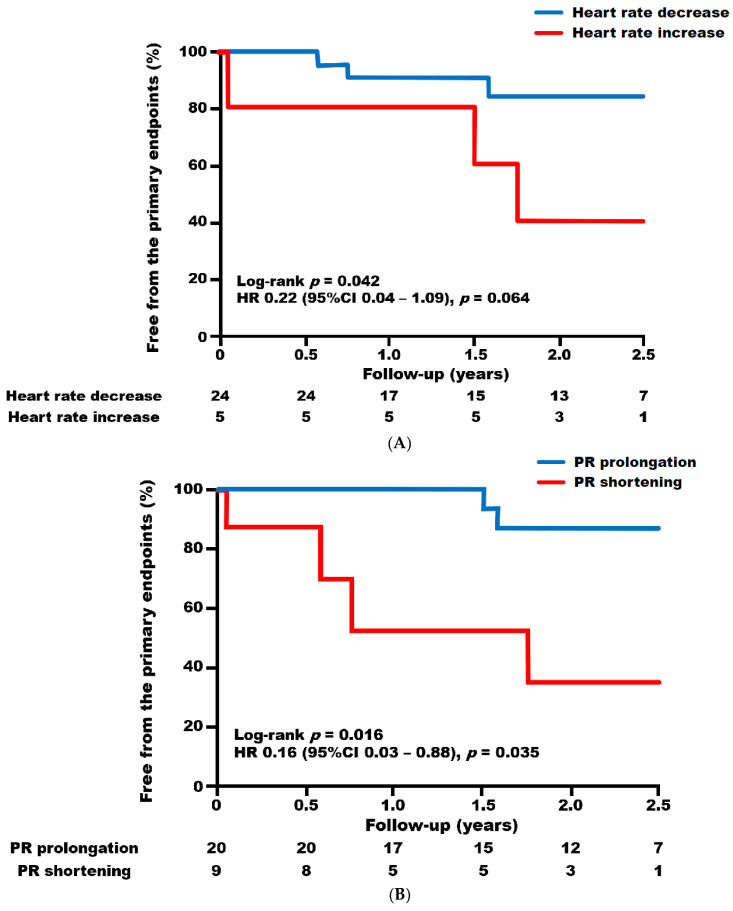

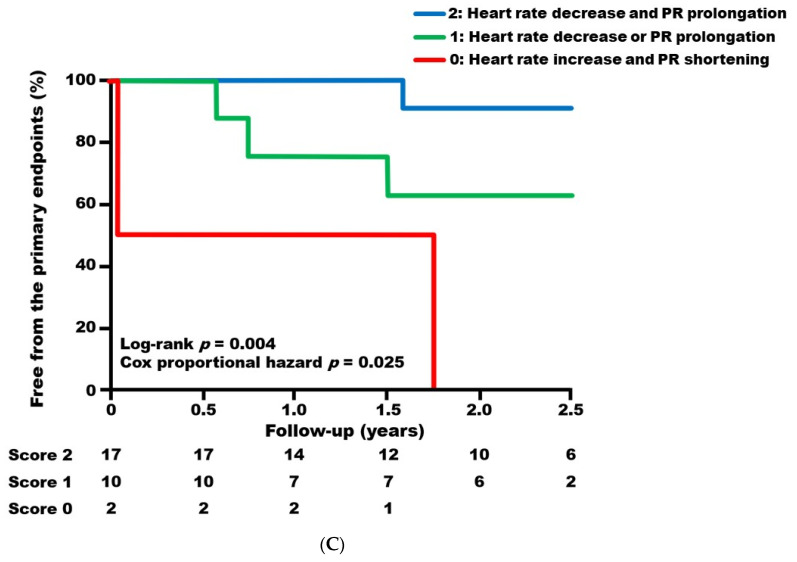
Kaplan–Meier survival curves stratified by the alteration of heart rate and PR interval one month following ivabradine administration; (**A**) Heart rate; (**B**) PR interval; (**C**) The combined scoring of heart rate increase and PR shortening, each assigned one point.

For the comparison of prognosis stratified by heart rate and PR interval alterations, Kaplan–Meier curve analyses were employed (Figure 4). Patients who experienced a heart rate decrease following ivabradine administration demonstrated a lower incidence of the primary endpoint compared to those with a heart rate increase (Log-rank *p* = 0.042, Figure 4A); however, the Cox proportional hazard model analysis did not yield statistical significance (*p* = 0.064). Patients who experienced PR prolongation demonstrated a lower incidence compared to those with PR shortening (Log-rank *p* = 0.016, hazard ratio of 0.16 [0.03–0.88], *p* = 0.035, Figure 4B). Notably, the combined scoring of heart rate decrease and PR prolongation, each assigned one point, effectively discriminated the incidence of the primary endpoint (Log-rank *p* = 0.004, Figure 4C).

The results of univariable analysis for predicting the outcomes comprising the primary endpoint are presented in Table 2. The only associated factor for the endpoint was the QRS width at baseline. After adjusting for QRS width, PR prolongation emerged as the independent predictor of favorable outcomes. The combination of a heart rate decrease and PR prolongation following ivabradine administration also demonstrated a favorable predictive value when compared with subjects who either did not satisfy either criterion or did not satisfy both criteria in this cohort (Table 3).

## 4. Discussion

### 4.1. Major Findings

To the best of our knowledge, this represents the initial report assessing alterations in the PR interval following ivabradine administration and the clinical implications of such changes in real-world clinical practice. The novel findings of this study are summarized as follows: (1) A predominant occurrence of PR prolongation accompanied by a decrease in heart rate was observed following ivabradine administration, (2) the PR interval exhibited a positive correlation with the fusion interval between trans-mitral E- and A-waves at baseline, and negative correlations with the truncated interval of the A-wave both before and after administration, and (3) the attainment of both heart rate reduction and PR prolongation following one-month ivabradine administration emerged as an independent predictor of favorable outcomes.

### 4.2. Impact of Ivabradine on Electrophysiology

The effects of ivabradine on the sinoatrial node have been thoroughly assessed [12]. I_f_ block-mediated reductions in heart rate have demonstrated diminished left ventricular remodeling and reduction in vascular oxidative stress markers in murine models [13,14]. In contemporary medical practice, ivabradine has been established as an alternative option for reducing heart rate in cases of persistently elevated heart rate despite the maximal dose of β-blockers administration [12].

On the contrary, little is known about the effects of ivabradine on the atrioventricular node in clinical practice. In an anesthetized mice model, ivabradine infusion demonstrated QRS widening and rate-dependent atrioventricular nodal conduction delay [15]. Our results revealed PR prolongation but not widening of P- and QRS-waves after ivabradine administration, suggesting that it does not affect conduction in the working myocardium of both the atrium and ventricle at clinical dosages. In healthy humans, the PR interval extends along with a decrease in heart rate [7]. However, the prolongation of the PR interval after ivabradine administration was independent of the reduction in heart rate in our study (Figure 2D). Furthermore, the PR interval adjusted by heart rate was significantly higher after administration than before, suggesting that ivabradine-induced atrioventricular conduction delay exceeded the physiological response to heart rate in clinical practice (Figure 2E). Regarding the repolarization phase, it has been established that the QT interval is not affected by ivabradine, consistent with our findings [16].

### 4.3. Impact of Ivabradine on Cardiac Conduction System

The prolongation of the PR interval, especially in the context of a first-degree atrioventricular block, is commonly recognized as a risk factor for atrial fibrillation, hospitalization, and mortality in patients with systolic heart failure [17]. However, PR prolongation might not always be associated with worse outcomes. For instance, it is not solely a first-degree atrioventricular block but rather the combination of a first-degree atrioventricular block with a prolonged QRS width that has been linked to mortality in a previous registry [18]. Certainly, QRS widening was correlated with unfavorable outcomes in our findings (Table 2), aligning with previous reports [18]. However, our data demonstrated that the majority fell within the normal range of QRS width.

Also, a significant contribution of P-wave duration to the PR interval was associated with a heightened risk of increased mortality, irrespective of whether the PR interval was short or long [19]. However, our results revealed that the interval of (PR–P-wave) was prolonged after ivabradine administration with no prolongation of P-wave duration (Figure 2C). This finding suggested that ivabradine contributes to the delay of atrioventricular nodal conduction without intra-atrial and intra-ventricular conduction disorders. These characteristics may contribute to understanding the results wherein PR prolongation, commonly recognized as a risk factor, was associated with favorable outcomes in our study. Furthermore, the majority of the cut-off values stratifying adverse events were set at PR intervals greater than 200 msec [20]. However, our results revealed that the majority of PR intervals did indeed remain shorter than 200 milliseconds (Figure 2B).

### 4.4. Impacts of PR Prolongation on Hemodynamics

The association between PR prolongation and hemodynamic parameters is another point of discussion. Our group previously reported that ivabradine-induced heart rate reduction contributes to the attenuation of the fusion between E- and A-waves. Reducing heart rate through either ivabradine or beta-blockers contributes to the decreased mortality observed in heart failure [21]. Furthermore, the E- and A-waves fusion has been established as the prognostic marker in patients with heart failure [22]. However, the previous study did not address the truncated A-wave and the PR interval [8].

Not only heart rate but also the PR interval has been recognized as a contributor to hemodynamics, especially in trans-mitral flow. In the present study, the interval of E- and A-waves fusion following ivabradine administration no longer exhibited a correlation with heart rate or the PR interval. However, the truncated interval of the A-wave consistently showed negative correlations with the PR interval both before and after administration. Given these results, situations involving a substantially lowered heart rate due to ivabradine may no longer present concerns regarding the fusion of E- and A-waves. Instead, they might manifest challenges associated with a truncated A-wave.

**Figure 5:**
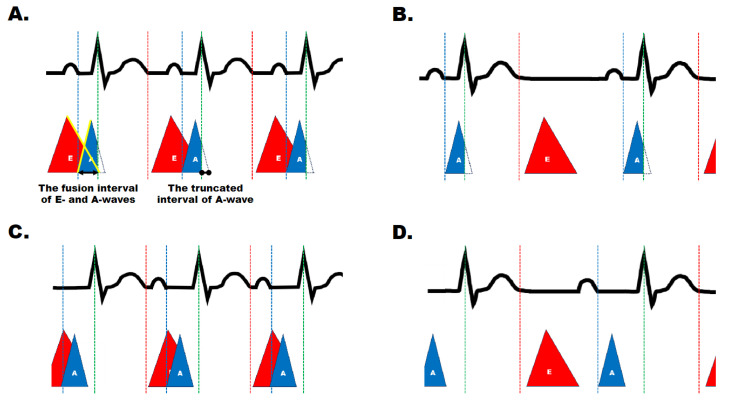
The hypothesis regarding the contribution of ivabradine to trans-mitral flow; (**A**) Rapid heart rate and short PR interval, indicating before ivabradine administration; (**B**) Slow heart rate without PR prolongation; (**C**) Rapid heart rate with PR prolongation; (**D**) Slow heart rate with PR prolongation, indicating following ivabradine administration. The top row indicates the surface electrocardiograms, and the bottom row represents the trans-mitral flow. Red triangles represent E-waves; blue, the A-wave; white, a truncated A-wave. Red dashed lines indicate the onset of the diastolic phase; blue lines, the onset of the atrial kick; green lines, the timing of atrioventricular valve closure.

Consequently, the PR prolongation induced by ivabradine could be linked to favorable outcomes in this study. In our interpretation, the potential hemodynamic superiority following ivabradine administration may be as follows (Figure 5). In the pre-ivabradine administration state, an inappropriately rapid heart rate results in the fusion of the terminal E-wave with the initial A-wave. Furthermore, a short PR interval leads to the truncation of the terminal A-wave due to the closure of the mitral valve (Figure 5A).

Heart rate decrease without PR prolongation (sub-optimal result):

A decrease in heart rate without PR prolongation results in a separation between E- and A-waves; however, a truncated A-wave still persists, which may result in impaired left ventricular filling (Figure 5B).

Heart rate increase with PR prolongation (sub-optimal result):

On the other hand, PR prolongation without a reduction in heart rate leads to further exacerbation of the fusion interval between both waves, although a truncated A-wave disappears (Figure 5C). 

Heart rate decrease with PR prolongation (optimal result):

Consequently, an appropriate PR prolongation with a decrease in heart rate addresses the fusion of E- and A-waves and a truncated A-wave (Figure 5D). Consequently, it is anticipated that the PR interval, when considered in conjunction with the heart rate, will contribute to hemodynamic superiority, thereby serving as a reliable prognostic factor.

### 4.5. Clinical Perspective

In order to enhance the number of patients deriving benefits from ivabradine efficacy, more frequent attainment of PR prolongation is necessary. The observed PR shortening following ivabradine administration might indicate an increased activity of the sympathetic nervous system. In cases of atrial fibrillation, ivabradine reduced the average or minimum ventricular rate but did not affect the maximum ventricular rate, indicating that ivabradine could not suppress atrioventricular conduction during heightened sympathetic nerve activity compared with vagal nerve activity [5]. Therefore, patients who did not exhibit PR interval prolongation following ivabradine administration may indicate over-activity of the sympathetic nerve. In such cases, consideration should be given to increasing the dosage of β-blockers to maximize the effects of ivabradine, as recommended in previous reports [23]. A slight increase in blood pressure by incremental cardiac output following administration of ivabradine would give us a further chance to up-titrate beta-blockers [24]. The potentially beneficial effects of PR interval-guided beta-blocker up-titration for recipients of cardiac resynchronization therapy have been reported [25].

Furthermore, the potential mechanisms contributing to PR prolongation induced by ivabradine include not only the expression of the If channel on the atrioventricular node but also the activation of vagal tone [26]. It is anticipated that these combined factors will exert an influence on the PR interval during the chronic phase following ivabradine administration, thereby contributing to favorable outcomes.

## 5. Limitations

The present study has several limitations. Firstly, the sample size was restricted in the single-center registry, leading to statistically limited power, particularly when adjusted for potential multi-cofounders such as anti-heart failure drugs. To validate these results, multicenter randomized trials are necessary. Secondly, the well-accepted correlation between heart rate and the fusion of E- and A-waves, which is generally acknowledged, was not demonstrated in this study. The results may have been influenced by the sample size. Notably, the PR interval demonstrated a negative correlation with the interval of the truncated A-wave, despite the limited sample size. Therefore, we contend that the impact of ivabradine on the PR interval is a robust predictor for prognosis. Finally, autonomic nerve activities could not be assessed in this study. The potential benefits of increasing the dosage of β-blockers for individuals experiencing PR shortening following ivabradine administration should be evaluated in future prospective randomized studies.

## 6. Conclusions

Ivabradine affected the PR interval during sinus rhythm in heart failure patients with systolic dysfunction. PR prolongation, along with a decrease in heart rate following ivabradine administration, was identified as an independent predictor of favorable outcomes. These results may be attributed to hemodynamic changes reflected in the trans-mitral flow pattern.

## Figures and Tables

**Table 1:** Clinical characteristics.

Variable	Overall (n = 29)
Demographics	
Age (years)	66 [52–76]
Male, n (%)	18 (62)
BMI (kg/m^2^)	21.8 [18.4–24.3]
Comorbidities	
Hypertension, n (%)	15 (52)
Diabetes mellitus, n (%)	11 (38)
Dyslipidemia, n (%)	15 (52)
Hemodialysis, n (%)	3 (10)
Etiology	
Ischemia, n (%)	12 (41)
Dilated cardiomyopathy, n (%)	9 (31)
Valvular heart disease, n (%)	3 (10)
Others, n (%)	5 (17)
Medications	
ACEi, ARB, or ARNI, n (%)	25 (86)
β-blockers, n (%)	24 (83)
Mineralocorticoid receptor antagonists, n (%)	21 (72)
Sodium-glucose transport protein 2 inhibitors, n (%)	10 (34)
Administration of the above four medical therapies, n (%)	8 (28)
Loop diuretics, n (%)	17 (59)
Tolvaptan, n (%)	11 (38)
Blood pressure	
Systolic, mmHg	103 [90–110]
Diastolic, mmHg	66 [58–75]
Baseline echocardiographic parameters	
Left atrial diameter, mm	31 [22–47]
Left ventricular ejection fraction, %	38 [34–49]
Mitral E-wave speed, cm/sec	30 [26–33]
Deceleration time to E-wave, msec	185 [124–237]
Mitral A-wave speed, cm/sec	64 [57–70]
Mitral E/A ratio	0.9 [0.6–1.3]
Interval of E- and A-waves fusion, msec	68 [33–103]
Laboratory data	
Serum creatinine, mg/dL	0.8 [0.7–1.6]
Estimated GFR, ml/min/1.73 m^2^	58.3 [35.7–78.4]
Plasma BNP, pg/mL	280 [137–606]
Serum NT-proBNP, pg/mL	1596 [999–4870]
Serum sodium, mmol/L	139 [138–142]
Serum potassium, mmol/L	4.2 [4.0–4.6]
Hemoglobin, g/dL	12.0 [11.1–14.0]

ACE, angiotensin-converting enzyme inhibitor; ARB, angiotensin receptor blocker; ARNI, angiotensin receptor–neprilysin inhibitor; BMI, body mass index; BNP, B-type natriuretic peptide; GFR, glomerular filtration rate; NT-proBNP, N terminal proBNP.

**Table 2:** Cox proportional hazard model analysis for predicting the primary endpoint.

	HR	95% CI	*p* Value
Age (1 year increase)	1.02	0.96–1.09	0.507
Male	3.02	0.35–25.94	0.313
BMI (1 kg/m^2^ increase)	1.01	0.82–1.19	0.954
Comorbidities			
Hypertension	1.22	0.24–6.04	0.811
Diabetes mellitus	4.68	0.85–25.71	0.076
Dyslipidemia	0.96	0.19–4.74	0.957
Hemodialysis	1.74	0.20–14.94	0.615
Etiology			
Ischemia	0.55	0.11–2.73	0.462
Medications			
Administration of the four drugs for heart failure	1.30	0.24–7.09	0.764
Loop diuretics	1.37	0.25–7.53	0.714
Vital signs before administration			
Heart rate (1 beat per minute increase)	0.98	0.94–1.03	0.427
Systolic blood pressure (1 mmHg increase)	1.00	0.97–1.00	0.687
Diastolic blood pressure (1 mmHg increase)	0.98	0.92–1.00	0.604
Baseline electrocardiographic parameters			
PR interval (1 msec increase)	0.98	0.95–1.01	0.134
QRS width (1 msec increase)	1.02	1.00–1.05	0.026
Corrected QT interval (1 msec increase)	0.99	0.98–1.01	0.238
Baseline echocardiographic parameters			
Left atrial diameter (1 mm increase)	1.03	0.94–1.12	0.553
Left ventricular ejection fraction (1% increase)	1.03	0.98–1.09	0.234
Mitral E-wave speed (1 cm/sec increase)	0.97	0.94–1.00	0.105
Deceleration time to E-wave (1 msec prolongation)	1.00	0.99–1.00	0.472
Mitral A-wave speed (1 cm/sec increase)	1.00	0.97–1.00	0.661
Interval of E- and A-waves fusion (1 msec increase)	1.00	0.99–1.01	0.604
Laboratory data			
Creatinine (1 mg/dL increase)	1.01	0.51–1.47	0.958
Estimated GFR (1 mL/min/1.73 m^2^ increase)	1.01	0.98–1.04	0.638
BNP (1 pg/mL increase)	1.00	1.00–1.00	0.715
NT-proBNP (1 pg/mL increase)	1.00	1.00–1.00	0.993
Sodium (1 mmol/L increase) iPotassium, mmol/L Hemoglobin, g/dLTroponin I, (1 pg/mL increase)	0.94	0.67–1.30	0.712
Potassium (1 mmol/L increase)	1.13	0.18–4.31 0.18	0.890
Hemoglobin (1 g/dL increase)	1.04	0.70–1.58	0.829

CI, confidence interval; HR, hazard ratio. Guideline-directed medical therapy refers to the administration of angiotensin converting enzyme inhibitors, angiotensin receptor blockers, or angiotensin receptor–neprilysin inhibitors, beta blockers, mineralocorticoid receptor antagonists, and sodium-glucose transport protein 2 inhibitors. Other abbreviations are the same as Table 1.

**Table 3:** Multivariable analysis for predicting the primary outcomes.

	Adjusted for QRS Width		
	HR	95% CI	*p* Value
Heart rate decrease	0.23	0.04–1.17	0.076
PR prolongation	0.06	0.01–0.67	0.022
Heart rate decrease and PR prolongation			
vs. heart rate increase or PR shortening	0.06	<0.01–0.69	0.025
vs. heart rate increase and PR shortening	0.01	<0.01–0.21	0.004

Abbreviations are the same as Table 2.

## Data Availability

The datasets used for this study are not publicly available but are available from the corresponding author on adequate request.

## Abbreviations

bpm	beats per minute
I_f_	funny current
msec	milliseconds
